# Successful transfer to sulfonylureas in *KCNJ11* neonatal diabetes is determined by the mutation and duration of diabetes

**DOI:** 10.1007/s00125-016-3921-8

**Published:** 2016-03-31

**Authors:** Tarig Babiker, Natascia Vedovato, Kashyap Patel, Nicholas Thomas, Roisin Finn, Roope Männikkö, Ali J. Chakera, Sarah E. Flanagan, Maggie H. Shepherd, Sian Ellard, Frances M. Ashcroft, Andrew T. Hattersley

**Affiliations:** Department of Diabetes and Endocrinology, Royal Devon and Exeter NHS Foundation Trust, Exeter, UK; Institute of Biomedical and Clinical Science, University of Exeter Medical School, Exeter, EX2 5DW UK; Department of Physiology, Anatomy and Genetics, University of Oxford, Parks Road, Oxford, OX1 3PT UK; UCL Institute of Neurology, MRC Centre for Neuromuscular Diseases, London, UK; Department of Diabetes and Endocrinology, Royal Sussex County Hospital, Brighton and Sussex University Hospitals, Brighton, UK

**Keywords:** ATP-sensitive potassium channel, Neonatal diabetes, Sulfonylurea receptor

## Abstract

**Aims/hypothesis:**

The finding that patients with diabetes due to potassium channel mutations can transfer from insulin to sulfonylureas has revolutionised the management of patients with permanent neonatal diabetes. The extent to which the in vitro characteristics of the mutation can predict a successful transfer is not known. Our aim was to identify factors associated with successful transfer from insulin to sulfonylureas in patients with permanent neonatal diabetes due to mutations in *KCNJ11* (which encodes the inwardly rectifying potassium channel Kir6.2).

**Methods:**

We retrospectively analysed clinical data on 127 patients with neonatal diabetes due to *KCNJ11* mutations who attempted to transfer to sulfonylureas. We considered transfer successful when patients completely discontinued insulin whilst on sulfonylureas. All unsuccessful transfers received ≥0.8 mg kg^−1^ day^−1^ glibenclamide (or the equivalent) for >4 weeks. The in vitro response of mutant Kir6.2/SUR1 channels to tolbutamide was assessed in *Xenopus* oocytes. For some specific mutations, not all individuals carrying the mutation were able to transfer successfully; we therefore investigated which clinical features could predict a successful transfer.

**Results:**

In all, 112 out of 127 (88%) patients successfully transferred to sulfonylureas from insulin with an improvement in HbA_1c_ from 8.2% (66 mmol/mol) on insulin, to 5.9% (41 mmol/mol) on sulphonylureas (*p* = 0.001). The in vitro response of the mutation to tolbutamide determined the likelihood of transfer: the extent of tolbutamide block was <63% for the p.C166Y, p.I296L, p.L164P or p.T293N mutations, and no patients with these mutations successfully transferred. However, most individuals with mutations for which tolbutamide block was >73% did transfer successfully. The few patients with these mutations who could not transfer had a longer duration of diabetes than those who transferred successfully (18.2 vs 3.4 years, *p* = 0.032). There was no difference in pre-transfer HbA_1c_ (*p* = 0.87), weight-for-age *z* scores (SD score; *p* = 0.12) or sex (*p* = 0.17).

**Conclusions/interpretation:**

Transfer from insulin is successful for most *KCNJ11* patients and is best predicted by the in vitro response of the specific mutation and the duration of diabetes. Knowledge of the specific mutation and of diabetes duration can help predict whether successful transfer to sulfonylureas is likely. This result supports the early genetic testing and early treatment of patients with neonatal diabetes aged under 6 months.

**Electronic supplementary material:**

The online version of this article (doi:10.1007/s00125-016-3921-8) contains peer-reviewed but unedited supplementary material, which is available to authorised users.

## Introduction

Neonatal diabetes is a monogenic subtype of diabetes which presents within the first 6 months of life [1]. A major cause of neonatal diabetes is an activating mutation in the *KCNJ11* gene, which encodes the inwardly rectifying potassium channel Kir6.2, which serves as the pore of the ATP-sensitive potassium (K_ATP_) channel. These mutations impair the ability of the channel to close in response to metabolically generated ATP, thereby preventing glucose-induced insulin secretion from pancreatic beta cells. Prior to identification of the genetic cause, neonatal diabetes patients with a *KCNJ11* mutation were treated with insulin. Subsequently, most have now transferred to sulfonylurea drugs, which directly close K_ATP_ channels and facilitate insulin release in response to food. This results in improved glycaemic control, fewer hypoglycaemic events and a simpler medication regime [2, 3].

A minority of individuals, however, are unable to fully stop insulin treatment [2]. A recent study of 58 patients suggested that the duration of diabetes prior to transfer correlated with the maintenance dose of sulfonylurea after transfer [4]. Other studies have suggested that the extent to which mutant channels are blocked by sulfonylureas in vitro predicts whether transfer is possible [5]. Thus, the aim of this study was to explore the effects of a wide range of *KCNJ11* mutations, and of diabetes duration, in a large sample (*n* = 127) of patients who attempted to transfer to sulfonylureas from insulin.

## Methods

### Patients

We retrospectively analysed clinical data on 127 patients from across the world (electronic supplementary material [ESM] Table 1) with mutations in the *KCNJ11* gene, as sequenced by the Molecular Genetics Laboratory, University of Exeter Medical School, UK. Data were collated from patients’ clinical records. For each patient, we noted the specific *KCNJ11* mutation, date of birth, sex, date of diagnosis, date of initiation of sulfonylurea, insulin dose (if applicable), sulfonylurea dose and HbA_1c_ level before and 4–12 months after sulfonylurea initiation. Weight before or within a year of transfer was converted to weight-for-age *z* scores (weight SD score) [6].

### Transfer to sulfonylureas

Patients with *KCNJ11* mutation and neonatal diabetes were transferred to sulfonylureas under the supervision of their local clinicians, using the Exeter protocol [7]. We defined successful transfer as a patient who could stop taking insulin altogether [2]. Unsuccessful transfer was defined as a patient who remained on insulin, either alone or in combination with a sulfonylurea, having attempted a dose of at least 0.8 mg/kg/day glibenclamide for at least 4 weeks (or the equivalent) [2]. When patients were prescribed a different sulfonylurea, the dose was calculated as a percentage of the maximum dose (according to the British National Formulary [www.bnf.org/]) and converted to an equivalent dose of glibenclamide, as described elsewhere [2]. All patients were treated with insulin before genetic diagnosis. None of the patients, including those unable to transfer, was prescribed any medication other than insulin and/or sulfonylureas to treat their diabetes.

### Functional analysis

The sulfonylurea sensitivity of recombinant wild-type and mutant pancreatic beta cell K_ATP_ channels (composed of Kir6.2 and sulphonylurea receptor 1 [SUR1] subunits) was measured in vitro using tolbutamide, as previously described [2]. Briefly, channels were expressed in *Xenopus* oocytes and whole-cell currents recorded by a two-electrode voltage clamp. The heterozygous state was simulated by co-injecting *ABCC8* mRNA (encoding SUR1) with 50:50 wild-type and mutant *KCNJ11* mRNA. Currents were activated by metabolic inhibition by azide (3 mmol/l) and the percentage block in response to tolbutamide (0.5 mmol/l; a maximally effective dose for wild-type channels) was measured.

### Statistical analysis

Clinical data were analysed using the Mann–Whitney *U* test and expressed as the median and interquartile range. Analysis was performed using Stata version 13.1 (StataCorp, College Station, Texas, USA).

### Ethics statement

This study was not deemed to require research ethics committee approval under Governance Arrangements for Research Ethics Committees guidelines because it involved research undertaken by staff within a care team using information collected in the course of care of their patients, which was anonymised for this study.

## Results

### Most patients with diabetes due to a *KCNJ11* mutation can transfer to sulfonylureas with improved glycaemic control

A total of 127 patients attempted transfer to sulfonylureas, and reached a dose of at least 0.8 mg/kg/day glibenclamide or an equivalent dose of another sulfonylurea. Of these, 112 (88%) transferred successfully and achieved excellent glycaemic control with sulfonylureas alone. In all, 15 patients failed to respond and remained on either insulin alone or insulin combined with a sulfonylurea. In patients who successfully transferred the median HbA_1c_ level fell from 8.2% (66 mmol/mol) on insulin to 5.9% (41 mmol/mol) after 4–12 months on sulphonylureas (*p* = 0.001).

### In vitro and in vivo sulfonylurea responses are strongly affected by the specific *KCNJ11* mutation

**Fig. 1:**
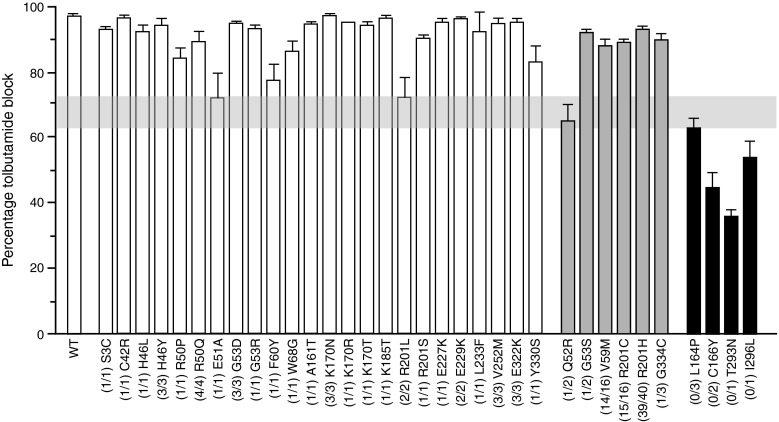
Sulfonylurea block of wild-type and mutant K_ATP_ channels. Percentage inhibition of whole-cell K_ATP_ channel currents in wild-type (WT) and mutant (as indicated) channels in response to 0.5 mmol/l tolbutamide. Current in the presence of tolbutamide is expressed as a percentage of that in the absence of drug [2, 9, 11–14]. The numbers in parenthesis indicate the number of patients who responded to sulfonylureas (left) out of the total number (right) with the indicated mutation. White bars, all patients responded; grey bars, some patients responded; black bars, no patients responded. The vertical light grey bar indicates the level of block that separates those patients who responded from those who did not

Figure 1 shows the extent of in vitro sulfonylurea block of wild-type channels and a range of mutant K_ATP_ channels vs the number of patients with each mutation who did or did not successfully transfer to sulfonylurea therapy. There is a clear correlation between the extent of block and the ability to transfer. No patients with the p.C166Y, p.I296L, p.L164P or p.T293N mutations (associated with a tolbutamide block of <63%) transferred. In contrast, most individuals with mutations associated with a tolbutamide block of >73% were able to transfer.

### A shorter diabetes duration is associated with successful transfer to insulin independence

**Fig. 2:**
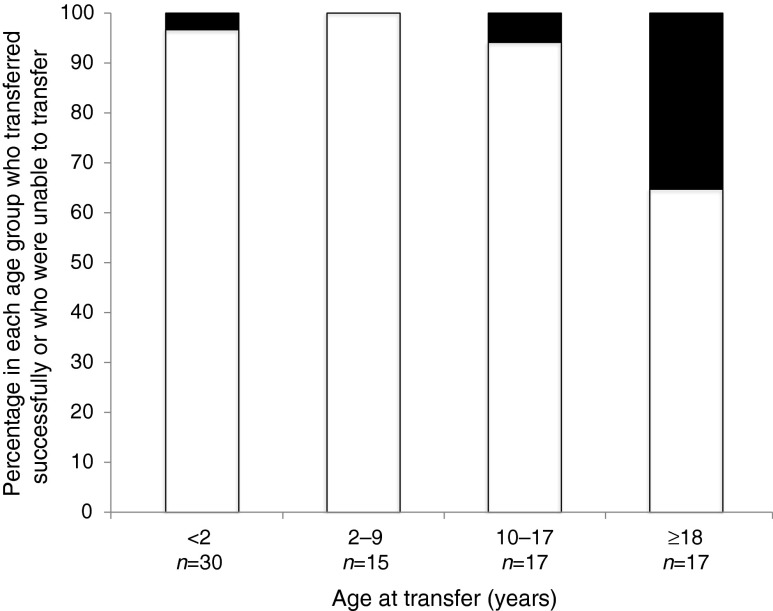
The younger the patients are (i.e. the shorter the duration of diabetes), the greater the likelihood of a successful transfer. Only *KCNJ11* mutations for which some patients transfer and others do not (p.R201H, p.R201C, p.V59M, p.G334C, p.G53S, p.Q52R) are shown. White bars, successful transfer; black bars, unsuccessful transfer

For six *KCNJ11* mutations (p.R201H, p.R201C, p.V59M, p.G334C, p.G53S and p.Q52R), some patients transferred to sulfonylureas whereas others did not (Fig. 1, grey bars). Those who successfully transferred had a shorter duration of diabetes prior to transfer compared with those who did not (mean 3.4 years [interquartile range (IQR) 0.3–11.9] vs 18.2 years [IQR 16.2, 18.9]; *p* = 0.032; Fig. 2).

Multivariate analysis showed that duration of diabetes (OR 0.92 [95% CI 0.86, 0.98], *p* = 0.008) and percentage tolbutamide block (OR 1.2 [95% CI 1.1, 1.3], *p* < 0.001) were independent and additive predictors of successful transfer to sulfonylureas.

The transfer success rate in patients with *KCNJ11* mutations was not associated with sex (*p* = 0.17), weight SD score at the time of transfer (*p* = 0.12) or the median HbA_1c_ level prior to attempted transfer (*p* = 0.87; ESM Table 2).

## Discussion

Most patients (88%) with neonatal diabetes due to a *KCNJ11* mutation could switch from insulin to sulfonylurea therapy and achieve near-normal glycaemia (HbA_1c_ 5.9% [41 mmol/mol]). However, our data show that successful transfer was influenced by both the specific mutation and the duration of diabetes.

All patients with the p.L164P, p.C166Y, p.T293N and p.I296L mutations failed to transfer to sulfonylurea therapy. This may be explained by the greatly reduced ability of sulfonylureas to block the mutant K_ATP_ channels. Sulfonylureas produce K_ATP_ channel inhibition by stabilising the long closed state of the channel [8]. The p.L164P, p.C166Y, p.T293N and p.I296L mutations dramatically decrease the time that the channel spends in this state, and thereby greatly reduce sulfonylurea inhibition [5, 9]. It is therefore very unlikely that patients with these mutations could transfer to sulfonylureas even if the dose were greatly increased. Therefore, a K_ATP_ channel blocker with a different mechanism of action is required.

For most mutations, all patients could transfer to sulfonylureas (Fig. 1, white bars; ESM Table 3). However, in each patient group, the numbers involved were relatively small. Thus, it is possible that later studies may identify some individuals with these mutations who fail to transfer. However, this will not be a consequence of a failure of sulfonylureas to block the K_ATP_ channel.

Of special interest are those mutations for which some patients transferred and others failed to do so despite taking an adequate dose of sulfonylureas. These include the most common mutations (p.R201H, p.R201C and p.V59M). Among these individuals, those who commenced sulfonylureas in the 5 years after diabetes diagnosis were very likely to successfully transfer. Recent recognition that diabetes before age 6 months is neonatal diabetes [1] and the availability of rapid molecular genetic diagnosis means that almost all patients should now be diagnosed with *KCNJ11* diabetes within weeks of diagnosis, thus facilitating a very early transfer.

One possible explanation for the better outcome when sulfonylureas are commenced at an earlier stage is that the patient’s beta cells have been exposed to chronic hyperglycaemia for a shorter period. In an inducible mouse model of neonatal diabetes carrying the *KCNJ11* p.V59M mutation, animals that were treated with glibenclamide shortly after gene induction showed normal islet cell morphology and excellent glycaemic control [10]. In contrast, mice exposed to chronic hyperglycaemia exhibited marked changes in insulin content and islet structure: these mice either required substantially more sulfonylurea to achieve normoglycaemia or sulfonylurea therapy was ineffective. This suggests that early initiation of sulfonylureas, and thus less exposure of beta cells to hyperglycaemia/hypoinsulinaemia, is important for successful transfer. In addition, it is possible that developmental changes influence beta cell function and that these are adversely affected by K_ATP_ channel overactivity or hyperglycaemia/hypoinsulinaemia.

In six individuals, the insulin dose was reduced by >50% but could not be discontinued completely. In these patients, we strongly support continuing both sulfonylurea and insulin. However, if there is no measurable C-peptide and no change in insulin dose when sulfonylurea treatment is used, then the only reason to continue sulfonylurea therapy is if beneficial effects on central nervous system function are observed. If this is not seen after a trial of at least 3 months, then sulfonylureas can be discontinued.

This study shows that the specific *KCNJ11* mutation and its in vitro response to tolbutamide is a crucial determinant of whether a patient will be able to transfer to sulfonylureas from insulin. For patients with responsive mutations, sulfonylurea transfer is less likely to succeed when it is attempted decades after diabetes diagnosis. This result emphasises the importance of obtaining an early molecular genetic diagnosis of *KCNJ11* neonatal diabetes because this provides information on whether transfer from insulin is likely to be successful and enables its rapid implementation.

## Electronic supplementary material

Below is the link to the electronic supplementary material.

ESM Table 1 (PDF 25 kb)

ESM Table 2 (PDF 36 kb)

ESM Table 3 (PDF 31 kb)

## Abbreviations

K_ATP_ channel: ATP-sensitive potassium channel
Kir6.2: Potassium inward rectifier 6.2
SUR1: Sulfonylurea receptor 1
