# Supplementary material for: Successful transfer to sulfonylureas in KCNJ11 neonatal diabetes is determined by the mutation and duration of diabetes
Source: Diabetologia. 2016 Mar 31;59:1162–6. doi: 10.1007/s00125-016-3921-8 (PMC4869695; doi:10.1007/s00125-016-3921-8)
Supplement: Supplementary file 1 — (PDF 25 kb) [file 125_2016_3921_MOESM1_ESM.pdf]

**Country**	**Number of patients**
Argentina	5
Australia	9
Austria	1
Bangladesh	1
Belgium	2
Brazil	1
Bulgaria	2
Canada	9
Chile	3
Croatia	1
France	1
Germany	5
Hungary	2
India	4
Ireland	2
Japan	1
Jordan	1
Latvia	1
Lebanon	1
Macedonia	1
Malaysia	1
Netherlands	2
New Zealand	1
Poland	4
Portugal	1
Puerto Rico	1
Romania	1
Russia	1
Saudi Arabia	1
Slovakia	3
South Africa	1
Sri Lanka	1
Switzerland	2
Turkey	3
UK	31
USA	19
Vietnam	1
**Total**	**127**

ESM Table 1. Countries of origin for the study
