# Supplementary material for: Successful transfer to sulfonylureas in KCNJ11 neonatal diabetes is determined by the mutation and duration of diabetes
Source: Diabetologia. 2016 Mar 31;59:1162–6. doi: 10.1007/s00125-016-3921-8 (PMC4869695; doi:10.1007/s00125-016-3921-8)
Supplement: Supplementary file 2 — (PDF 36 kb) [file 125_2016_3921_MOESM2_ESM.pdf]

**Clinical characteristics in p.R201H, p.R201C, p.V59M, p.G334C, p.G53S, p.Q52R combined i.e. mutations where some, but not all patients transfer**	**Successful transfer (n=71)**	**Unsuccessful transfer (n=8)**	***p* value**
Prior to transfer			
Duration of diabetes (years)	3.4 (0.3, 11.9)<br>n=69	18.2 (16.2, 18.9)<br>n=6	0.032
HbA1c (%) prior to transfer [mmol/mol]	8.5 (7.3, 9.3)<br>[69 (56,78)]<br>n=51	8.0 (7.6, 11.2)<br>[64 (60, 99)]<br>n=4	0.87
Weight SDS prior to transfer or within 12 months of transfer	-0.65 (-1.38, 0.15)<br>n=37	1.45 (-1.19, 2.45)<br>n=4	0.12
Sex (%)	Female 49%<br>n=35	Female 75%<br>n=6	0.17
After sulfonylurea was started			
HbA1c (%) 4-12 months after transfer [mmol/mol]	5.9 (5.4, 6.5)<br>[41 (36, 48)]<br>n=45	8.2 (6.5, 8.2)<br>[66 (48, 66)]<br>n=6	0.0047
Dose of sulfonylurea (mg/kg/day) 4-6 months after first started SU	0.6 (0.3, 0.7)<br>n=32	1.0 (1.0, 2.0)<br>n=5	0.0029

ESM Table 2. Characteristics of individuals with neonatal diabetes due to the mutations where some, but not all patients are able to transfer successfully to sulfonylureas. All characteristics except sex are expressed as medians with interquartile ranges.
