# Supplementary material for: Successful transfer to sulfonylureas in KCNJ11 neonatal diabetes is determined by the mutation and duration of diabetes
Source: Diabetologia. 2016 Mar 31;59:1162–6. doi: 10.1007/s00125-016-3921-8 (PMC4869695; doi:10.1007/s00125-016-3921-8)
Supplement: Supplementary file 3 — (PDF 31 kb) [file 125_2016_3921_MOESM3_ESM.pdf]

**Clinical characteristics**	**Mutations where all patients transfer (n=41)**	**Mutations where no patients transfer (n=7)**	***p* value**
Prior to transfer			
Duration of diabetes (years)	0.8 (0.3, 11.4)<br>n=40	5.3 (1.3, 17.1)<br>n=6	0.41
HbA1c (%) prior to transfer<br>[mmol/mol]	7.7 (6.7, 8.8)<br>[61 (50, 73)]<br>n=25	8.4 (7.7, 8.7)<br>[68 (61, 72)]<br>n=4	0.68
Weight SDS prior to transfer	-0.58 (1.37, 0.29)<br>n=12	-3.58 (-4.87, 0.40)<br>n=3	0.25
Sex (%)	Female 41%<br>n=17	Female 71%<br>n=5	0.15
After sulfonylurea was started			
HbA1c (%) 4-12 months after transfer<br>[mmol/mol]	6.1 (5.6, 6.6)<br>[43 (38, 49)]<br>n=25	8.7 (7.7, 9)<br>[72 (61, 75)]<br>n=3	0.01
Dose of sulfonylurea (mg/kg/day) 4-6 months after first started SU	0.5 (0.2, 0.6)<br>n=9	1.0<br>n=1	0.12

ESM Table 3. Characteristics of individuals with neonatal diabetes where all individuals within a mutation transfer to sulfonylureas (p.A161T, p.C42R, p.E227K, p.E229K, p.E322K, p.E51A, p.F60Y, p.G324R/G324R, p.G53D, p.G53R, p.H46L, p.K170N, p.K170R, p.K170T, p.K185T, p.L233F, p.Q52L, p.R201L, p.R201S, p.R50P, p.R50Q, p.S3C, p.V252M, p.W68G, p.Y330S) compared to where all individuals within a mutation fail to transfer (p.C166Y, p.I296L, p.L164P, p.T293N). All characteristics except sex are expressed as medians with interquartile ranges.
